# Assessment of Human Intelligence—The State of the Art in the 2020s

**DOI:** 10.3390/jintelligence12080072

**Published:** 2024-07-27

**Authors:** Johanna M. deLeyer-Tiarks, Jacqueline M. Caemmerer, Melissa A. Bray, Alan S. Kaufman

**Affiliations:** 1 Psychology Department, Pace University, New York, NY 10038, USA; 2 Department of Educational Psychology, University of Connecticut, Storrs, CT 06268, USA

## 1. An Introduction to the Special Issue

Contemporary intelligence theory and assessment in the United States—a century after Lewis Terman published the *Stanford–Binet* in 1916—has evolved in ways that even David Wechsler could not have envisioned. The major aim of this Special Issue is to define the breadth and scope of cognitive assessment in the 2020s, from infancy to adulthood, as a testament to how far the field has advanced in the century since IQ testing was synonymous with *g*. This Special Issue attests to the array of instruments that have joined Wechsler’s and Terman’s scales in the clinician’s toolbox and the diversity of theories that have impacted cognitive assessment over the past generation that continue to be refined and redefined, including the innovative research that continually shapes the direction for future generations.

In assembling this Special Issue, we sought contributions from psychologists around the world—theoreticians, clinicians, researchers—who have been instrumental in defining the current state of the art and are leading the charge into the future of intellectual assessment. The esteemed authors in this Special Issue include legends in the field (Phillip Ackerman, Nancy Mather, Kevin McGrew, Samuel Ortiz, and Robert Sternberg) and an array of rising stars such as Danny Hajofsky, LaTasha Holden, and Joel Schneider. This Special Issue is greatly enhanced by the articles contributed from Greece (Sofologi et al. 2023), Germany (Rusche and Ziegler 2023), and Australia (Vaughan and Birney 2023).

## 2. Equity in Cognitive Assessment 

The early study of intelligence tests was steeped in prejudicial values which maintained the superiority of white, western culture. Today, research in the theory, development, implementation, and interpretation of cognitive testing has moved on from eugenics, *g* theory, and fixed intelligence, so that the landscape of modern assessment can evolve toward a framework that is more equitable and socially just, in concert with a society that increasingly emphasizes the importance of diverse viewpoints.

Several papers included in this Special Issue highlight biases inherent to intelligence tests due to the historical and empirical contexts in which they were developed. Ortiz and Cehelyk (2024) edify the contemporary issues in the assessment of multilingual individuals by discussing the role of culture and language development in cognitive assessment. They point to the need for tests to be developed with adequate validity and fairness for individuals who have not been raised in the language or culture of the predominant modern intelligence tests. Furthermore, this Special Issue includes an exploration of emergent, non-*g*, models of intelligence. Those such as process overlap theory (Holden and Tanenbaum 2023) allow for examination of individual strengths and weaknesses with an emphasis on parsimony in assessment, thus furthering researchers’ and practitioners’ work toward equity and fairness goals within the field of intelligence assessment.

## 3. Contemporary Use of Cognitive Assessment Results 

The intelligence tests used to inform diagnoses of specific learning disabilities, intellectual disabilities, developmental disorders, and giftedness are built upon a strong foundation of theory and empirical research. The goal of clinical, neuropsychological, and psychoeducational evaluation is to make a difference in the lives of the children and adults referred for evaluation. Test results and clinical observations are translated into action, often in the form of educational interventions.

This Special Issue includes articles that highlight the diverse applications of intelligence tests within society, across a broad age range, using tests that extend well beyond the Wechsler–Binet monopoly that dominated assessment a half-century ago. Winter et al. (2023) studied infant and toddler abilities within the cognitive, language, and motor domains—as measured by the *Bayley Scales of Infant and Toddler Development-4th edition* (Bayley-4)—to better understand the impact of being born prematurely on children’s functioning. Notably, these authors were able to identify significant differences within each domain based on the degree of prematurity and the age at which these young children were evaluated. Mather and Schneider (2023) provided a comprehensive discussion of the use of intelligence tests to diagnose youth with dyslexia, and Hajovsky et al. (2023) examined the Woodcock–Johnson tests to better understand the influence of youth’s general intelligence (*g*) on the predictive relation between their specific cognitive abilities and basic reading and reading comprehension skills.

## 4. Theoretical and Methodological Advancements in the Assessment of Intelligence 

Both the growth and acceleration of theory-based intelligence test development and interpretation have been geometric during the past three decades and continue their ascent. Furthermore, contemporary society in the U.S. has had no less of an impact on clinical research and practice than the latest theories or statistical procedures. In this Special Issue, McGrew and colleagues discuss methods, specifically psychometric network analysis, to further refine the widely used Cattell–Horn–Carroll theory of cognitive abilities (CHC theory) and enhance the field’s understanding of the CHC cognitive abilities (McGrew et al. 2023). Sternberg (2022) argues that, to account for the positive application of individuals’ intelligence for society’s success, contemporary theories of intelligence must consider the attitudinal component of intelligence.

In tandem with the evolution of intelligence theory, methods of intelligence testing are reimagined over time. Ackerman (2022) conceptualizes the modern use of process and content intelligence measures by presenting them within their historical context. To provide further insight into how intelligence testing can be best utilized, Vaughan and Birney (2023) describe and demonstrate the benefits of measuring the short-term variability in a person’s cognitive performance across time. In recent years, the procedures to measure individuals’ intelligence have shifted from in-person to remote assessment. Mulligan and Ayoub (2023) present a balanced perspective of the benefits and pitfalls of remote assessment practices. Lastly, Sofologi et al. (2023) describe how teachers’ metacognitive knowledge and practical intelligence influence their teaching practices.

## 5. Closing Remarks 

The contributions in this Special Issue promote a deeper understanding of theoretical and methodological advancements of cognitive assessment in the 2020s and offer a provocative peek into the future. These topics are relevant for researchers and clinicians who use intelligence tests and theory to support individuals’ and society’s success. We conclude this introduction to this Special Issue on a sad note. Dr. Marianna Massimilla Rusch, whose doctoral dissertation formed the basis of the article on domain-specific knowledge (Rusche and Ziegler 2023), passed away before the paper was published. We dedicate this Special Issue to her memory.
